# Global Genome Diversity and Recombination in *Mycoplasma pneumoniae*

**DOI:** 10.3201/eid2801.210497

**Published:** 2022-01

**Authors:** Yu-Chia Hsieh, Shiao-Wen Li, Yi-Yin Chen, Ching-Chia Kuo, Yin-Cheng Chen, Ian Yi-Feng Chang, Yi-Jiun Pan, Ting-Hsuan Li, Ruei-Lin Chiang, Ya-Yu Huang, Wei-Chao Liao

**Affiliations:** Chang Gung University, Taoyuan, Taiwan (Y.-C. Hsieh, S.-W. Li, Y.-C. Chen, I.Y.-.F. Chang, R.-L. Chiang, W.-C. Liao);; Chang Gung Children's Hospital, Taoyuan (Y.-C. Hsieh, Y.-Y. Chen, C.-C. Kuo, T.-H. Li, Y.-Y. Huang);; Linkou Chang Gung Memorial Hospital, Taoyuan (I.Y.-.F. Chang, W.-C. Liao);; China Medical University, Taichung, Taiwan (Y.-J. Pan)

**Keywords:** Mycoplasma pneumoniae, bacteria, recombination, global genome diversity, repetitive element, adaptation, evolution, Taiwan

## Abstract

Genomic changes in *Mycoplasma pneumoniae* caused by adaptation to environmental or ecologic pressures are poorly understood. We collected *M. pneumoniae* from children who had confirmed pneumonia in Taiwan during 2017–2020. We used whole-genome sequencing to compare these isolates with a worldwide collection of current and historical clinical strains for characterizing population structures. A phylogenetic tree for 284 strains showed that all sequenced strains consisted of 5 clades: T1–1 (sequence type [ST]1), T1–2 (mainly ST3), T1–3 (ST17), T2–1 (mainly ST2), and T2–2 (mainly ST14). We identified a putative recombination block containing 6 genes (MPN366‒371). Macrolide resistance involving 23S rRNA mutations was detected for each clade. Clonal expansion of macrolide resistance occurred mostly within subtype 1 strains, of which clade T1–2 showed the highest recombination rate and genome diversity. Functional characterization of recombined regions provided clarification of the biologic role of these recombination events in the evolution of *M. pneumoniae*.

*Mycoplasma pneumoniae*, a major pathogen causing community-acquired pneumonia, has a reduced genome of ≈800 kb, which has been relatively stable over time and geographic distance (*1*). Widespread use of macrolides has driven an increase and spread of macrolide-resistant *M. pneumoniae* in several countries, including Taiwan (*2**–**4*). High numbers of repetitive DNA elements (RepMPs), designated RepMP1, RepMP2/3, RepMP4, and RepMP5, comprise ≈8% of the *M. pneumoniae* genome and play essential roles in survival niches of *M. pneumoniae* by engaging in recombination events to generate surface antigen diversity (*5*). Epidemiologic data show that epidemic peaks of *M. pneumoniae* occur every 3‒7 years, as indicated by 3 outbreaks in 2011‒2012, 2014‒2015, and 2015‒2016 in Japan and Europe (*6**,**7*). Genotype shifts from 1 P1 adhesin subtype to another occurred repeatedly at an interval of 10 years (*7*).

The relationship between epidemic periodicity and genotype shifts is unclear. P1 adhesin causes antigenic variation between clinical strains as a result of homologous recombination between RepMP2/3 and RepMP4 domains located within their open reading frames and at repetitive DNA elements at other sites in the bacterial genome (*8*). However, P1 adhesin type is not always the determinant factor for cyclic outbreaks because cocirculation of both P1 subtypes and multiple variants in endemic and epidemic settings has been documented (*9*). Given the existence of multiple copies of specific RepMPs dispersed across chromosomes, whether other genetic regions showing recombination diversity involved in *M. pneumoniae* circulation remains unclear.

To study how the *M. pneumoniae* lineage has evolved as it has spread, we characterized the genomes of 99 *M. pneumoniae* strains isolated in Taiwan, together with a global collection of 185 *M. pneumoniae* genomes deposited in public databases. At the same time, we sought to identify major putative recombination hot spots on a genome wide scale to comprehensively understand the significance of recombination on the evolutionary dynamics of *M. pneumoniae*.

## Materials and Methods

### Isolation of *M. pneumoniae*

We prospectively enrolled children <18 years of age who were hospitalized because of clinical and radiographic pneumonia during April 2017‒January 2020 at Chang Gung Memorial Hospital Lin Kou Branch (Taoyuan, Taiwan), Chang Gung Memorial Hospital Kaohsiung Branch (Kaohsiung, Taiwan), and Saint Paul’s Hospital (Taoyuan, Taiwan). We obtained written informed consent from patients or their parents. Throat swab specimens were then collected by pediatricians who used sterile swabs (FLOQSwabs; Copan, https://www.copanusa.com). All throat swab specimens were sent to a laboratory at the Chang Gung Memorial Hospital Lin Kou Branch. We inoculated throat swab specimens into SP4 medium and observed spherical *M. pneumoniae* colonies by using microscopy. This study protocol was approved by the research ethics committee of Chang Gung Memorial Hospital, Taiwan (approval nos. 201900420A3 and 202000687B0).

### Whole-Genome Sequencing

We obtained whole-genome sequencing (WGS) data for 99 clinical isolates. We cultured *M. pneumoniae* in 75-cm^2^ tissue culture flasks (Techno Plastic Products AG, https://www.tpp.ch) containing 10 mL of SP4 medium for 96 hours at 37°C. We extracted *M. pneumoniae* genomic DNA directly from cultures by using the QIAamp DNA Mini Kit (QIAGEN, https://www.qiagen.com), and prepared libraries for WGS by using NEBNext Ultra II DNA Library Prep Kits (Illumina, https://www.illumina.com).

### Prediction of Recombination Sites by Phylogenetic Analysis

We combined 185 isolates that had available WGS data, downloaded from the RefSeq database of the National Center for Biotechnology Information (NCBI; https://www.ncbi.nlm.nih.gov), and our assembled 99 clinical isolates (Appendix 1 Table). We used all 284 *M. pneumoniae* isolates to analyze homologous recombination and genetic diversity as described (*10*).

### Clade-Specific Recombination Gene Sequence Analysis

We used Gubbins (https://sanger-pathogens.github.io/gubbins) to identify areas that were likely introduced by homologous recombination involving *M. pneumoniae* clades. We scored each gene with the number of predicted recombination events in each clade, and used R version 3.6.3 (R Foundation for Statistical Computing, https://cran.r-project.org) to draw a heatmap by using the pheatmap package.

### Estimation of Synonymous and Nonsynonymous Substitution Rates

We annotated 284 *M. pneumoniae* by using Prokka version 1.14.6 (*11*) and identified core genes by using Roary version 3.13.0 (*12*) with default settings. We aligned sequences of genes by using ClustalW2 (*13*) and converted protein and DNA multiple sequence alignments into codon alignments by using PAL2NAL (*14*). We used the output data file to estimate synonymous and nonsynonymous substitution rates (*K*_a_/*K*_s_) in different branches of the phylogenetic tree by using a branch-specific algorithm in the codeml program from PAML version 4.8 (*15*), for which we provide additional details (Appendix 2). We used M129 as a reference genome to describe the location of RepMPs based on the NCBI annotation file.

### Tip-Dating Analysis

We used whole-genome alignments to construct a phylogenetic tree by using RAxML (https://cme.h-its.org/exelixis/software.html) with the GTRCAT model. We performed root-to-tip regression analysis by using TempEst version 1.5.3 (*16*). To estimate the phylodynamics of *M. pneumoniae*, we performed temporal analysis using the BEAST version 1.10.4 software package (*17*). We used a strict clock exponential model and a Hasegawa–Kishono–Yano nucleotide substitution model with a discretized gamma distribution to measure rate heterogeneity across sites. We summarized tree data to generate a maximum clade credibility tree by using TreeAnnotator (https://beast.community/treeannotator#user-interface) and visualized it by using FigTree version 1.4.4 (*1*).

### Population Structure Analysis

We used PLINK version 1.9 (*18*) to generate a binary output from the vcf file. We performed population structure inference by using ADMIXTURE version 1.3.0 (*19*).

## Results

### Genotype Distribution

From the NCBI database, we collected *M. pneumoniae* strains from China (n = 23), Japan (n = 70), South Korea (n = 30), the United States (n = 29), the United Kingdom (n = 4), Denmark (n = 3), Spain (n = 1), France (n = 15), Germany (n = 2), Guatemala (n = 1), Egypt (n = 2), Kenya (n = 3), and Tunisia (n = 2) obtained during 1944‒2016 (Figure). These strains included P1 subtype 1 sequence type (ST) 3, prevalent worldwide; P1 subtype 2 ST2 from Japan, the United States, and countries in Europe; P1 subtype 1 ST1 from the United States, South Korea, China, and Tunisia; P1 subtype 1 ST17 from South Korea; P1 subtype 2 ST7 from Japan; and P1 subtype 2 ST14 from South Korea, Japan, France, and the United States.

**Figure:**
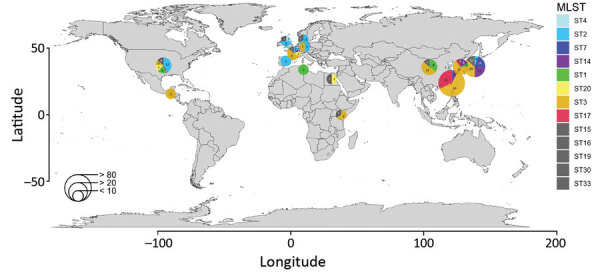
Genotype and origin of *Mycoplasma pneumoniae* genome data compared in study of global genome diversity and recombination. Pie charts indicate number of isolates in indicated countries. Detailed information regarding each sample is provided in Appendix 1 Table 1. MLST, multilocus sequence typing; ST, sequence type.

We observed macrolide resistance with the A2063G mutation among ST3 strains from China, South Korea, Japan, and France; ST1 strains from China; ST7 strains from Japan; and ST14 strains from South Korea and Japan. A total of 99 strains from Taiwan collected during 2017‒2020 consisted of P1 subtype 1 ST3 (n = 60, 60.6%), P1 subtype 1 S17 (n = 31, 31.3%), P1 subtype 2 ST14 (n = 7, 7.1%), and P1 subtype 2 ST2 (n = 1, 1%). Macrolide resistance with A2063G was also observed in ST3 (n = 59), ST17 (n = 14), and ST14 (n = 3). A2063T was observed in ST17 (n = 10), A2064G in ST3 (n = 1).

### Recombination Landscape

We inferred genomic regions identified by Gubbins as having densities of single-nucleotide variants (SNVs) that were different from background SNV densities in the core genome to be homologous recombination events. By examining all 284 *M. pneumoniae* genomes in this study, we detected 108 putative recombination blocks (Appendix 2 Figure 1, panel A) spanning an average of 1.3 kb/recombination event (95% CI 0.9–1.7 kb) and covering an average of 10 kb/isolate (1.3% of the genome).

Phylogenetic analysis using core genome single-nucleotide polymorphisms (SNPs) identified 5 clades linked to multilocus sequence typing structures and associated with P1 subtypes, which resembled previous findings (*1**,**7**,**20*). Clade T1–1 consisted of ST1; clade T1–2 consisted of ST3, ST19, ST20, and ST30; clade T1–3 consisted of ST17; clade T2–1 consisted of ST2, ST4, ST7, and ST16; clade T2–2 consisted of ST2, ST14, ST15, and ST33. Clades T1–1, T1–2, and T1–3 belonged to P1 subtype 1, and clades T2–1 and T2–2 belonged to P1 subtype 2.

With regard to switching from subtype 1 to subtype 2, two regions of SNPs were predicted to have arisen by homologous recombination: first, 183717–186553 bp sequence coordinates containing P1 adhesin (MPN141) for T2–1 and 183381–187741 bp for T2–2; and second, 437080–443621 bp sequence coordinates containing hypothetical proteins (MPN366–367), RepMP1, DUF16 domain-containing protein (MPN368), lipoprotein (MPN369), putative adhesin (MPN370), and MPN371 for both clades T2–1 and T2–2. Among strains of subtype 1, clade T1–2 had 3 regions of SNPs predicted to have arisen by homologous recombination: first 34,0697–340757 bp sequence coordinates containing *prrB* (MPN285); second, 493781–493791 bp sequence coordinates containing adhesin (MPN409); and third, 570767–570790 bp sequence coordinates containing adhesin (MPN468) (Appendix 1 Table 2; Appendix 2 Figure 1, panel A).

The MPN409 recombined region was more frequently detected in most macrolide-resistant clade T1–2 strains than in macrolide-susceptible clade T1–2 strains. We ran Gubbins independently for subtype 2 (88 isolates) based on the core genome sequence alignment involving *M. pneumoniae* subtype 2 reference strain FH (GenBank accession no. CP010546.1). Among strains of subtype 2, only 1 recombination hotspot was predicted: 179477–183466 bp sequence coordinates containing P1 adhesin (equal to *MPN141* of M129) (Appendix 1 Table 2; Appendix 2 Figure 1, panel B).

We estimated 2 measures of the recombination rate in the core genome alignment by using Gubbins ρ/θ and r/m ratios (Table). We provide boxplots of putative recombination statistics for *M. pneumoniae* clades (Appendix 2 Figure 2). ρ/θ estimates the relative frequency of occurrence of recombination and mutation in the history of the clade. The overall ρ/θ ratio for each clade ranged from 0 (clade T2–1) to 0.03 (clade T1–2), indicating that putative recombination events have occurred less frequently than vertically inherited mutations in all 5 clades, and lower than in most microbes. The r/m index assesses the relative effect of recombination and mutation on genetic diversification of the clade. Predicted recombination occurred more frequently in clade T1–2, for which the r/m rate was 4–13 times higher than for the other 4 clades.

**Table:** Putative recombination statistics for *Mycoplasma pneumoniae* clades for study of global genome diversity and recombination*

Clade	No. strains	Total SNPs	No. SNPs inside recombination	No. SNPs outside recombination	No. recombination blocks	r/m	ρ/θ
T1–1	17	10.29 ± 23.44	5.65 ± 20.62	4.65 ± 6.78	0.18 ± 0.53	0.03 ± 0.14	0.01 ± 0.02
T1–2	146	9.78 ± 12.41	1.76 ± 4.06	8.02 ± 10.88	0.24 ± 0.55	0.25 ± 0.91	0.03 ± 0.12
T1–3	33	3.00 ± 5.70	0.18 ± 1.04	2.82 ± 5.53	0.03 ± 0.17	0.04 ± 0.21	0.01 ± 0.03
T2–2	46	14.46 ± 13.65	0.33 ± 1.71	14.13 ± 13.28	0.04 ± 0.21	0.02 ± 0.07	0 ± 0.01
T2–1	42	12.93 ± 16.71	1.98 ± 5.13	10.95 ± 12.59	0.26 ± 0.63	0.07 ± 0.17	0.01 ± 0.02

*Values are mean ± SD. r/m assesses the relative effect of recombination and mutation on genetic diversification of the clade. ρ/θ estimates the relative frequency of occurrence of recombination and mutation in the history of the clade. SNP, single-nucleotide polymorphism.

### Clade-Specific Gene Recombination

We defined clade-specific genes involved in these putative recombination events by clustering of genes by recombination frequency in each clade (Appendix 2 Figure 3, panel A). These data can guide identification of multiple genes under different selective pressures in each clade. A set of 8 genes (*MPN141, MPN142, MPN366, MNP368, MPN369, MPN370, MPN_RS02085*, and *MPN_RS02055*) appeared to increase the recombination frequency in *M. pneumoniae* subtype 2, and extensive DNA repetitive elements are located in each of these genes. *MPN366, MPN368, MPN369*, and *MPN370* were under even greater selective pressure than *MPN141* and *MPN142* (Appendix 2 Figure 3, panel A).

We investigated adhesin P1 sequence variation by calculating homology with the reference M129 strain DNA sequence (Appendix 2 Figure 3, panel B). Two variable regions provided most variation between *M. pneumoniae* subtypes 1 and 2, and were located at ≈3000 bp and ≈1000 bp within *MPN141*. In contrast, these 2 regions were highly conserved within clades T1–1, T1–2, and T1–3. For subtype 2, clade T2–2 had less conserved sequence than clade T2–1. 

Two additional regions of nucleotide sequence differed among strains belonging to clade T2–2 compared with M129. Sequence analysis of the P1 gene showed divergent sequences from 5 clades at position 2000 bp, which might be an AGT trinucleotide variable-number tandem repeat (*21*).

### Genome Diversity within Subclades

To clarify this potentially confounding within‐clade mutation, we analyzed the genetic diversity of the collection by estimating the nonsynonymous to synonymous (*K*_a_/*K*_s_) mutation ratio. We assessed divergence along the genome between clades by determining synonymous substitution rates, a near-neutral indicator of genetic divergence. Data showed that different genomic regions had higher divergence among those 5 clades. Clade T2–1 had more divergent regions than clade T2–2, and clade T2–2 had higher *K*_s_ values in less divergent regions. The genomic divergence pattern of clade T1–2 was observed across the genome. This result indicated that clade T1–2 was the most divergent in *M. pneumoniae* subtype 1, and the genome of clade T1–3 was stable without evidence of divergence (Appendix 2 Figure 4, panel A). We also identified numerous diverse regions located near RepMP elements across the genome. We further investigated the function of these genomic regions that showed higher divergence. From a global perspective, numerous positions are located in genes that are annotated as adhesin and MgPa (*mgp*) operon of *M. genitalium* coding for proteins (Appendix 2 Figure 4, panel B).

### Positive Selection Detection Involving Orthologous Genes

To track genomic evolutionary footprints, we used codeml analysis to test genes for evidence of positive selection. We detected 42 genes as being under positive selection by using likelihood ratio tests (Appendix 1 Table 3). Of these positively selected genes, 21 were core orthologous loci present among all 284 genomes investigated. To determine the relationship of genes with certain evolutionary features, we classified these genes into different functional categories by using clusters of orthologous groups of proteins annotation. There are a large number of genes in the functional categories translation, ribosomal structure, and biogenesis and cell wall/membrane/envelope biogenesis. The cell wall/membrane/envelope biogenesis category includes membrane-associated genes under positive selection, consistent with observations for other bacterial species (*22*). The translation, ribosomal structure, and biogenesis category, including ribosomal biogenesis genes, might be correlated with drug resistance.

### Evolution Timeline

We calculated the temporal history of *M. pneumoniae* by using Bayesian evolutionary analysis sampling trees to place the most recent common ancestor in ≈1300 (95% highest posterior density 1230–1392) by exponential growth analysis (Appendix 2 Figure 5). The maximum clade credibility tree also showed 2 distinct clades that are consistent with the 2 subtypes for P1 gene variation. This analysis suggested that the 2 subtypes might have diverged at approximately the same time as the most recent common ancestor arose. The evolutionary divergence time estimates for subtypes 1 and 2 for the 5 clades were from 1800 to 1900 (subtype 1, 1823–1865; subtype 2, 1811–1855). *M. pneumoniae* subtypes 1 and 2 spread rapidly within 20 years (through the year 2000).

### Population Structures and Sequences

Genetic history can be established by examining the patterns of shared genetic variation between >2 populations. Admixture proportion inference is an algorithm used to infer the proportions of ancestry from each source population based on shared genetic drift. These proportions can produce visual summaries that identify a population structure. We used ADMIXTURE to fit a model which the genome is composed of sites from *K* = 2 to *K* = 10 (*K* = n, where n is the number of ancestral generations) for ancestral populations. We provide ADMIXTURE analysis with estimated proportions of 7 ancestral populations (Appendix 2 Figure 6).

We provide unsupervised models assuming *K* = 4 and *K* = 7 ancestral clusters (Appendix 2 Figure 6, panel A). At *K* = 7, it is difficult to discern the major contributors to each clade, which is likely to result in a disrupted relationship between clades T2–1 and T1–1 and other clades. Because each column represents 1 isolate, we show data for admixed strains observed particularly in clades T1–2 and T2–2, reflecting the divergence of the 2 clades (Appendix 2 Figure 6, panel A).

To further clarify the genetic track, we mapped populations to geographic region (Appendix 2 Figure 6, panel B). Each pie chart shows the mean composition of 7 populations in each region. The distribution shows genetic diversity related to geography. Thus, *M. pneumoniae* has the potential to be transmitted efficiently and spread regionally from Europe to America, and subsequently emerge rapidly in Asia. Genome sequences generated during this study were deposited into the NCBI BioProject database (accession no. PRJNA699672).

## Discussion

According to our predictions, *M. pneumoniae*, a major cause of atypical bacterial pneumonia described in the 1930s, might have originated in Europe, and has existed for >200 years. Our analysis showed that *M. pneumoniae* genome evolution by recombination varies by clade, as well as recombined regions dependent on clades. In addition to the well-known P1gene locus, there are recombined regions focused around surface protein genes, which are believed to be targets of natural selection and are involved in affecting *M. pneumoniae* population structures. Adherence to cells of the respiratory tract is considered a prerequisite for colonization and pathogenesis by *M. pneumoniae*, which adhere to host target cells by a polar structure known as the attachment organelle (*23*). On the surface of this organelle, P1 and 2 accessory proteins P40/P90 form a transmembrane adhesion complex, which is directly involved in receptor binding (*23*).

Our clinical and epidemiologic information for *M. pneumoniae* is based primarily on typing of the P1 gene. Two major P1 genotype strains (1 and 2) represent evolutionarily divergent clades. In Japan, genotype shifts between P1 subtypes 1 and 2 driven by the human immune system repeatedly occurred at 10-year intervals with transitionary 2–3 years intervals during the past 40 years, including a recent shift from P1 subtype 1 in the 2000s to subtype 2 in the later 2010s (*7*). In this study, by comparing subtype strains 1 and 2, we identified a new predicted recombination block located on *MPN366‒371*. Apart from *MPN366* and *MPN367*, which were predicted as pseudogenes, MPN369 lipoprotein and MPN370 are predicted to be expressed as cell-surface proteins.

Theoretically, the increase in frequency of recombination involving these regions might be caused by selective advantages offered by divergent sequences introduced by putative recombination events. The biologic role of putative recombination events in *MPN366–371* is worthy of further investigation. These loci might be associated with antigenic variation and adherence to promote the persistent circulation of *M. pneumoniae* lineages in human populations.

RecA, single-stranded DNA-binding protein, RecU, RuvA, and RuvB are enzymes responsible for DNA recombination in *M. pneumoniae* based on sequence homology analysis (*24*). Sluijter et al. found that subtype 1 strains harbor a nonsense protein‒truncating codon in the *recU* gene. This gene is fully expressed in subtype 2 strains, but there is no detectable enzyme activity under various conditions (*24*). Given that there are more P1 subtype variants among strains of subtype 2 compared with strains of subtype 1 determined from epidemiologic data (*25*), RecU in subtype 2 strains might show enzyme activity under >1 cofactors (*24*).

We also found predicted recombination hot spots only in regions flanking the P1 gene but not in other genomic regions among strains of subtype 2. Among strains of subtype 1, we found hot spots in 3 non-P1 gene regions in clade T1–2 (*MPN285*, *MPN409*, and *MPN468*), despite no recombination events involving the P1 gene region. These 3 genes were predicted to express the type I restriction enzyme EcoKI-specificity protein, adhesin, and adhesin P1 family proteins, respectively. Clade T1–2 showed the highest apparent recombination rate compared with the other clades. The protein machinery required for DNA repeat sequence rearrangement might differ by clade, which would account for the observed dissimilar recombination kinetics involving repetitive genetic elements.

Since 2010, macrolide-resistant *M. pneumoniae* have caused several epidemics in Asia. Macrolide resistance involving point mutations in 23S rRNA occurred in each clade. However, in countries that had high rates of macrolide resistance, clonal expansion of ST3 was seen most commonly (*2*). Clade T1–2 (ST3 strains), which appeared during 1960, has predominantly circulated in Europe, the United States, and Asia. Our analysis showed that clade T1–2 had the highest *K_s_* value and greatest genome diversity, which should be associated with the reason why it became an epidemiologically successful genome worldwide even under the selective pressure exerted by macrolide overuse.

Overall, homologous recombination played only a minor role in shaping diversity within *M. pneumoniae*, based on the low ratio of r/m, estimated to be 0.05. However, the highest r/m ratio of clade T1–2 indicated that probable recombination between genetic repetitive elements across chromosomes makes greater contributions to clonal diversification in clade T1–2. Our phylogenetic analysis indicated an additional clade T1–3 (ST17 strains) compared with those obtained in a study in South Korea conducted by Lee et al. (*1*). ST17 is another smaller macrolide-resistant clone that spread in Taiwan (*4*). It is hypothesized that predominance of macrolide-resistant *M. pneumoniae* in subtype 1 is associated with macrolide overuse when subtype 1 is circulating (*7*). During 2017, subtype 2 replaced subtype 1 as the dominant subtype and had decreased macrolide resistance in Japan (*7*), but increased macrolide resistance was detected in China (*26*). It is imperative to continuously monitor spread and evolution of macrolide-resistant subtype 2 strains to obtain additional perspectives on macrolide resistance.

Although this study demonstrates the need for WGS for studying genetic diversity, the first limitation is that the sample size of the genome sequence for studying evolution of *M. pneumoniae* was small. Second, populations of *M. pneumoniae* can be found worldwide, but only 284 isolates were sequenced in our study, which represented only a few countries and lacked global representation, geographic distribution, and evolutionary spread between regions. Third, we predicted recombination events by using Gubbins, which identified loci containing elevated densities of base substitutions while concurrently constructing a phylogeny based on putative point mutations outside these regions. Homologous recombination identified in our study might be a hotspot of SNV occurrence by natural mechanisms. Because detection of recombination is challenging, these results only predict the possibility of recombination by using tools. However, these regions have the potential to undergo recombination events.

In summary, this study comprehensively demonstrated detailed insights into the recombination dynamics within *M. pneumoniae*. Further delineation of the role of homologous recombination in the virulence and adaptation of *M. pneumoniae* to modern environments will provide useful information for public health issues.

Appendix 1 Additional genetic information on global genome diversity and recombination in *Mycoplasma pneumoniae*.

Appendix 2 Additional information on global genome diversity and recombination in *Mycoplasma pneumoniae*.
