# Supplementary material for: Global Genome Diversity and Recombination in Mycoplasma pneumoniae
Source: Emerg Infect Dis. 2022 Jan;28(1):111–7. doi: 10.3201/eid2801.210497 (PMC8714221; doi:10.3201/eid2801.210497)
Supplement: Appendix 2 — Additional information on global genome diversity and recombination in Mycoplasma pneumoniae. [file 21-0497-Techapp-s2.pdf]

Article DOI: https://doi.org.10.3201/eid2801.210497

Global Genome Diversity and Recombination in *Mycoplasma pneumoniae*

Appendix 2

Supplementary Methods

Whole-Genome Sequencing

Library samples were multiplexed and sequenced on an Illumina (https://www.illumina.com) NextSeq 500 Platform with 2 × 150-bp paired-end reads. Adaptor and poor quality sequences were trimmed by using BBduk (*1*), and genomes were assembled de novo by using SPAdes version 3.13.0 (*2*) with k-mer values of 127 and in careful mode. This genome assembly, which was evaluated by using QUAST (*3*), produced an average of 18 contigs with an N50 length of 143,785 bp (Appendix 1, Table 4, https://wwwnc.cdc.gov/EID/article/28/1/21-0497-App1.xlsx). In silico multilocus sequence typing, a typing technique more discriminatory than multiple locus variable-number tandem repeat analysis and P1 typing (*4*), was performed by using a BLAST (https://blast.ncbi.nlm.nih.gov/Blast.cgi)–based tool (https://github.com/tseemann/mlst) on de novo genome assemblies (*5*).

Prediction of Recombination Sites by Phylogenetic Analysis

Variants were called by using the *Mycoplasma pneumoniae* M129 genome (GenBank accession no. NC_000912.1), a macrolide-sensitive sequence type 1 strain isolated in the United States during 1968, as reference by using Snippy version 4.4.5 (*6*), which uses BWA Mem (https://github.com/lh3/bwa) to map the reads or sequences to the reference, and then subsequently calls single-nucleotide polymorphisms and insertions/deletions (indels) with FreeBayes (https://github.com). Reference-based

alignments of the core genome output from Snippy (Snippy-Core) were used for downstream phylogenetic analysis. Recombination sites were assessed by using Gubbins version 2.3.4 (*7*), and a maximum-likelihood tree was generated by using RAxML version 8.2.10 (*8*). RAxML was implemented by using the GTRCAT model, with no rate heterogeneity or ascertainment bias, rapid hill climbing, and default parameters. After 4 iterations, Gubbins reached a stable tree topology, including inferred regions of hypothetical genetic recombination events. The resulting phylogenetic tree, isolate metadata, core genome single-nucleotide polymorphisms, and recombination sites were visualized by using Phandango version 1.3.0 (*9*).

Lineage-Specific Recombination Gene Sequence Analysis

In this study, to explore *MPN141* gene divergence, 148 isolates could be extracted from the *MPN141* sequence and aligned by using MUSCLE version 3.8.31 (*10*). Pairwise differences were calculated by comparison to the reference M129 stain and used a 25-bp sliding window with incremental 1-bp step sizes.

Estimation of Synonymous and Nonsynonymous Substitution Rates

Codon frequencies were fixed by using the F3X4 model and a branch-site alternative model (model = 2, NS sites = 2) with settings according to Huang et al. (*11*). To detect genes under positive selection, we applied the codeml algorithm to estimate a single ω for all sites in the alignment. Three selection models were generated with initial ω (K_a/K_s) values of 0.5, 1, and 1.5, and we fixed ω at 1 as the neutral model. To identify genes with significance, we performed likelihood ratio tests between the neutral model (M1) and selection models (M2) with adjusted p values <0.05. To visualize the distribution of K_s divergence as genome-wide views involving 5 lineages, we calculated the mean value of by K_s by using a 25-gene window.

Population Structure Analysis

To determine the ideal number of populations, we assumed K from 2 to 10, and calculated cross-validation and likelihood statistics. The K that yielded the smallest cross-validation value and the highest likelihood index was chosen to execute the admixture.

References

1. Bushnell B. BBMap [cited 2021 Sep 2]. https://sourceforge.net/projects/bbmap

2. Bankevich A, Nurk S, Antipov D, Gurevich AA, Dvorkin M, Kulikov AS, et al. SPAdes: a new genome assembly algorithm and its applications to single-cell sequencing. J Comput Biol. 2012;19:455–77. PubMed https://doi.org/10.1089/cmb.2012.0021

3. Gurevich A, Saveliev V, Vyahhi N, Tesler G. QUAST: quality assessment tool for genome assemblies. Bioinformatics. 2013;29:1072–5. PubMed https://doi.org/10.1093/bioinformatics/btt086

4. Brown RJ, Holden MT, Spiller OB, Chalker VJ. Development of a multilocus sequence typing scheme for molecular typing of *Mycoplasma pneumoniae.* J Clin Microbiol. 2015;53:3195–203. PubMed https://doi.org/10.1128/JCM.01301-15

5. Seemann T. Scan contig files against PubMLST typing schemes: tseemann/mlst, 2018 [cited 2021 Sep 2]. https://githubcom/tseemann/mlst

6. Li H, Durbin R. Fast and accurate short read alignment with Burrows-Wheeler transform. Bioinformatics. 2009;25:1754–60. PubMed https://doi.org/10.1093/bioinformatics/btp324

7. Croucher NJ, Page AJ, Connor TR, Delaney AJ, Keane JA, Bentley SD, et al. Rapid phylogenetic analysis of large samples of recombinant bacterial whole genome sequences using Gubbins. Nucleic Acids Res. 2015;43:e15. PubMed https://doi.org/10.1093/nar/gku1196

8. Stamatakis A. RAxML-VI-HPC: maximum likelihood-based phylogenetic analyses with thousands of taxa and mixed models. Bioinformatics. 2006;22:2688–90. PubMed https://doi.org/10.1093/bioinformatics/btl446

9. Hadfield J, Croucher NJ, Goater RJ, Abudahab K, Aanensen DM, Harris SR. Phandango: an interactive viewer for bacterial population genomics. Bioinformatics. 2018;34:292–3. PubMed https://doi.org/10.1093/bioinformatics/btx610

10. Edgar RC. MUSCLE: multiple sequence alignment with high accuracy and high throughput. Nucleic Acids Res. 2004;32:1792–7. PubMed https://doi.org/10.1093/nar/gkh340

11. Huang CL, Pu PH, Huang HJ, Sung HM, Liaw HJ, Chen YM, et al. Ecological genomics in *Xanthomonas*: the nature of genetic adaptation with homologous recombination and host shifts. BMC Genomics. 2015;16:188. PubMed https://doi.org/10.1186/s12864-015-1369-8

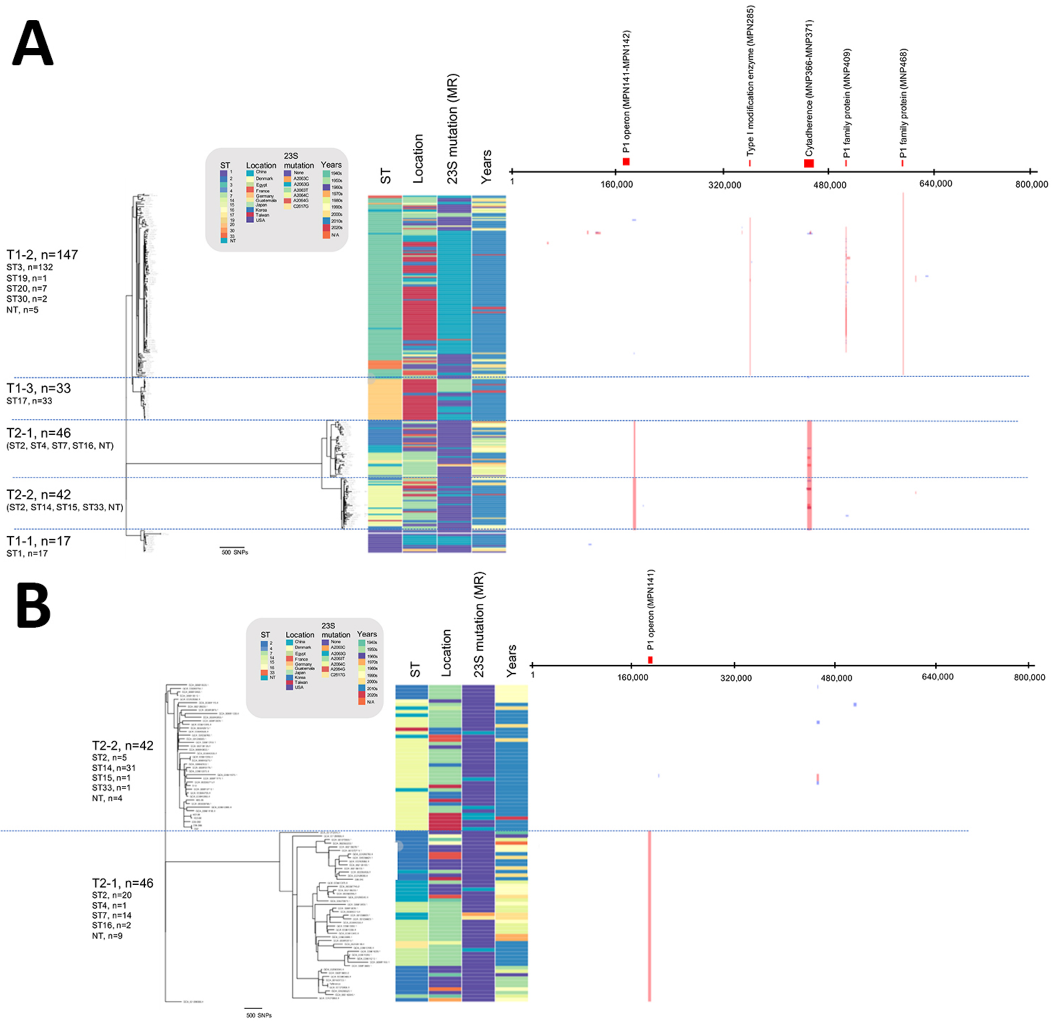

Appendix 2 Figure 1. A) Global recombination predictions for 284 *Mycoplasma pneumoniae* genomes. *M. pneumoniae* phylogeny based on single-nucleotide polymorphism density is shown on the left. Colored blocks represent putative recombination regions identified by using Gubbins software. Coordinates of recombination sites were based on those for strain M129. B) Putative recombination events detected in *M. pneumoniae* subtype 2. Gubbins analysis identified 1 region of predicted recombination (red lines) in clade T2–1 compared with strain FH across the genome.

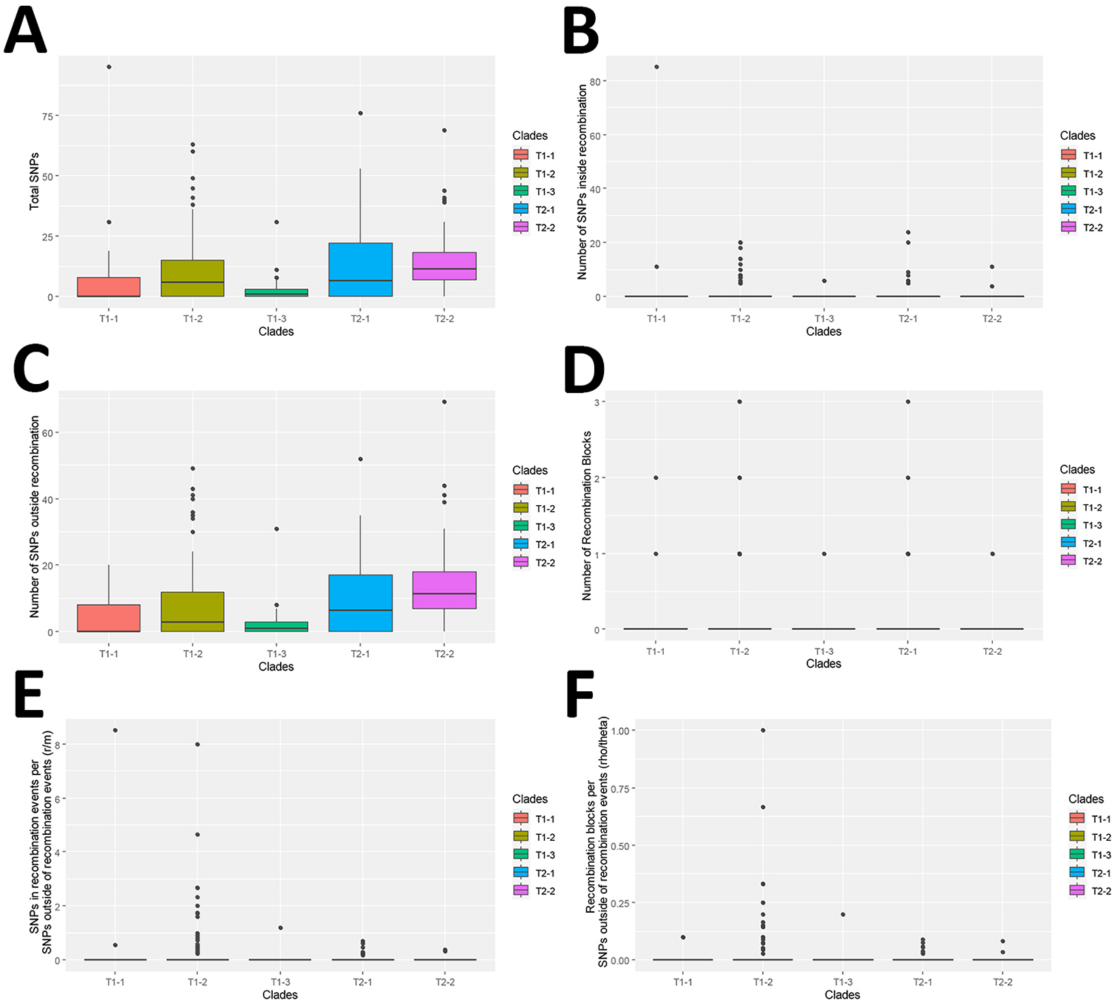
A
Total SNPs
Clades
T1-1
T1-2
T1-3
T2-1
T2-2
B
Number of SNPs inside recombination
C
Number of SNPs outside recombination
D
Number of Recombination Blocks
E
SNPs in recombination events per SNPs outside of recombination events (r/m)
F
Recombination blocks per SNPs outside of recombination events (rho/theta)

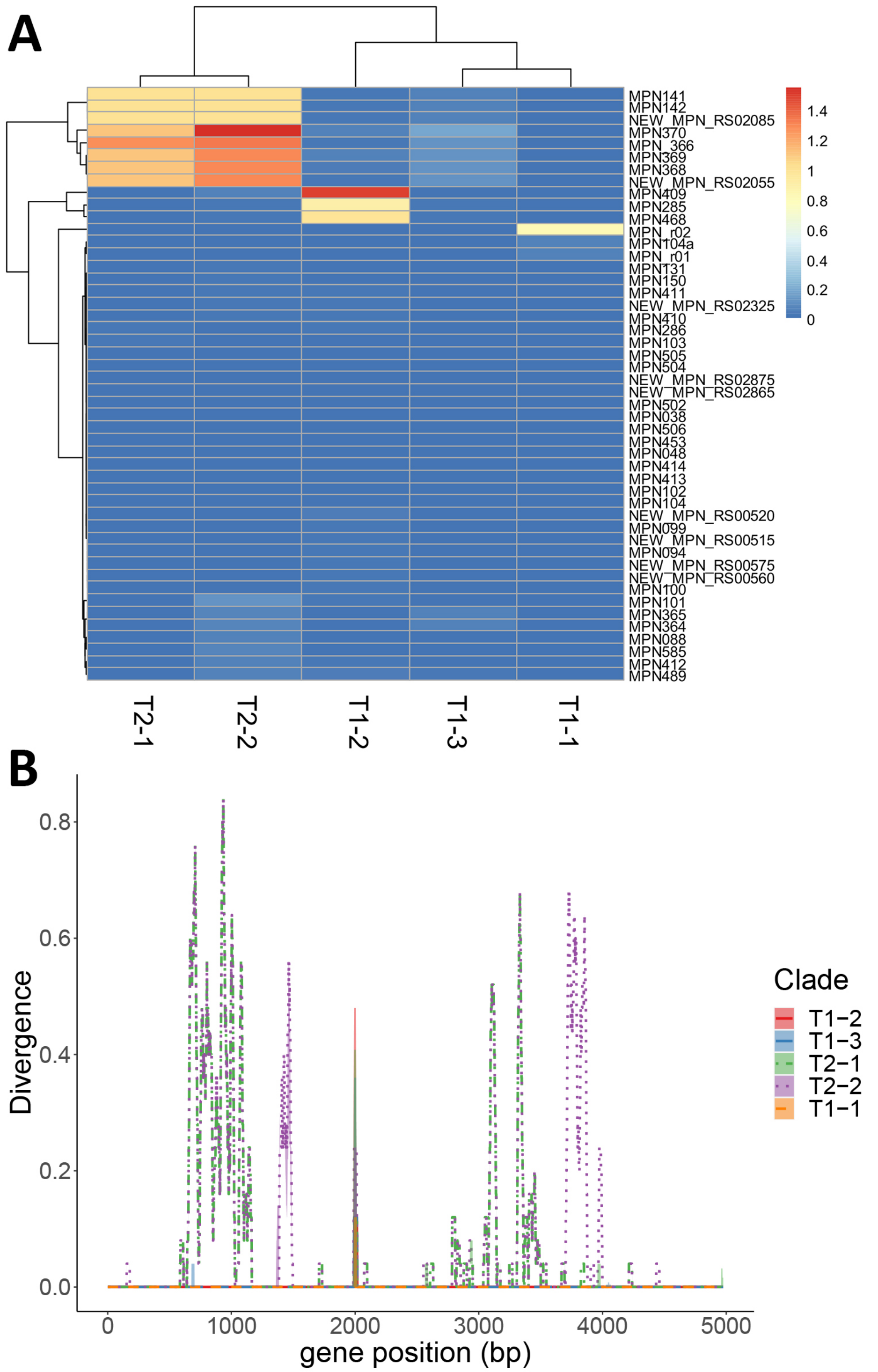

Appendix 2 Figure 3. A) Clade-specific recombination profiles at the gene level for *Mycoplasma pneumoniae*. Each horizontal line represents a gene, with colors corresponding to means of putative recombination events. B) Divergence (proportion of differing nucleotides) of the *MPN141* gene for each clade in 148 *M. pneumoniae* genomes compared with those for strain M129.

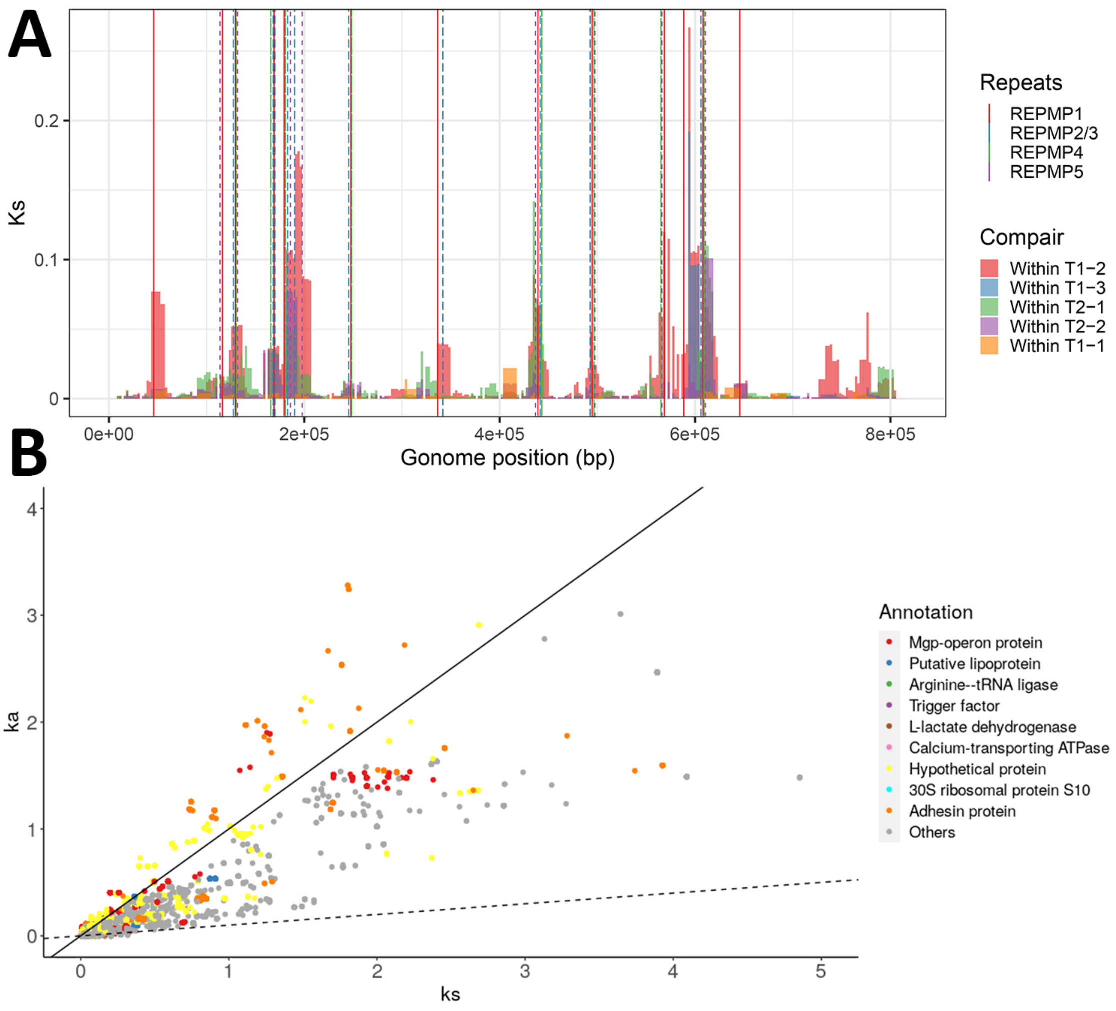

Appendix 2 Figure 4. Genome-wide variation among 284 *Mycoplasma pneumoniae* strains. A) Distribution of K_s divergence in M129 genomes with repeat elements. B) K_a/K_s distribution of 3,644,877 pair in 971 paralogs in 284 *M. pneumoniae* strains. Solid line indicates $K_a/K_s = 1$, and dotted line indicates slope = 0.01. The top 10 functions, which showed exhibited higher K_a mean values, are colored. Mgp, *M. genitalium*.

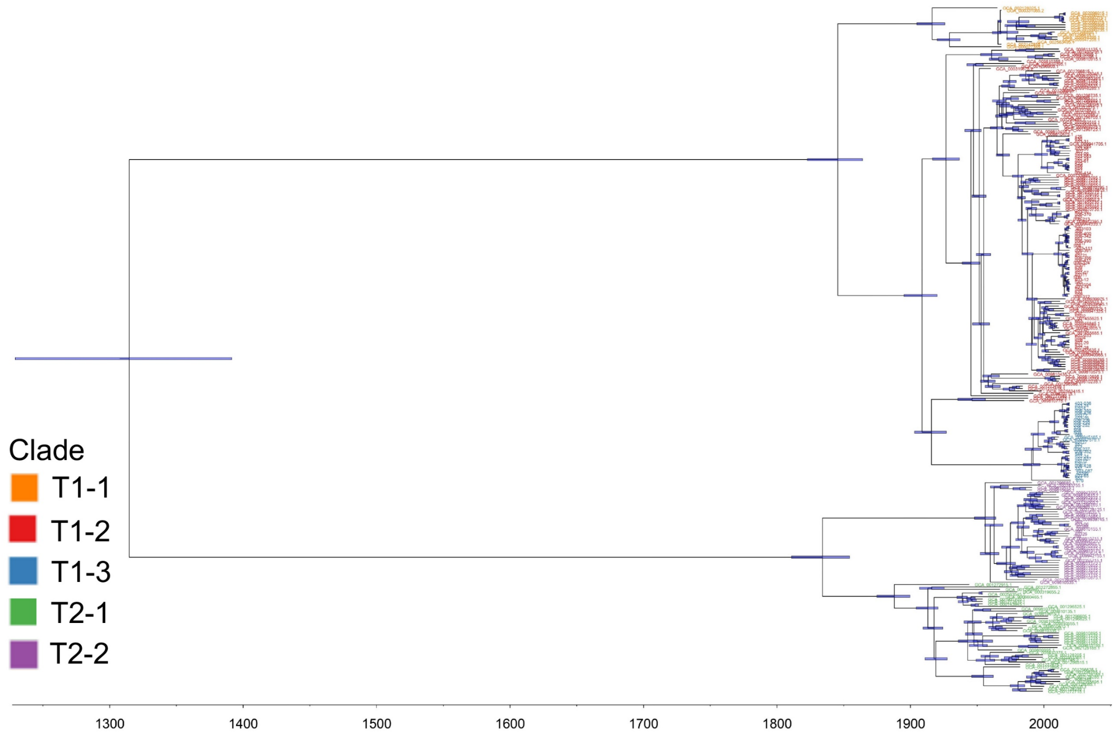

Appendix 2 Figure 5. Maximum-likelihood phylogenetic tree of aligned nucleotide sequences of *Mycoplasma pneumoniae* isolates. Dates along the x-axis are in years (CE), and blue bars indicate 95% posterior probabilities.

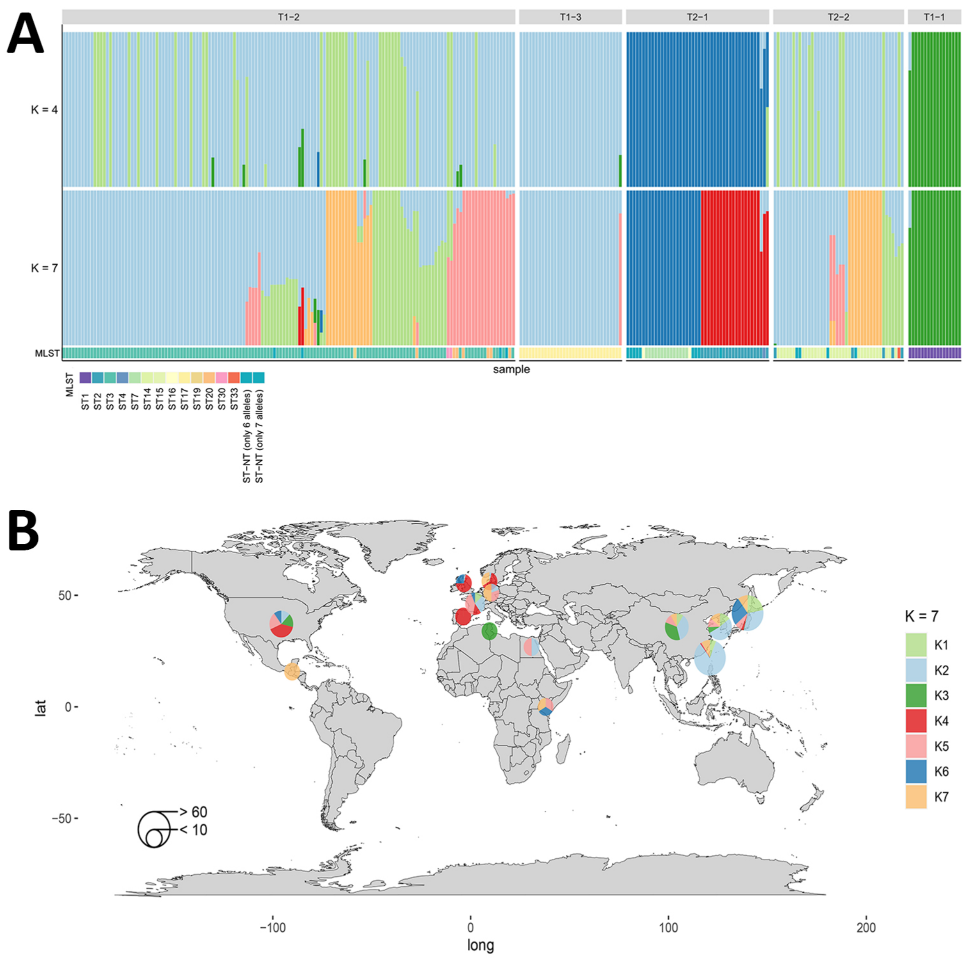

Appendix 2 Figure 6. Genetic structure and geographic analysis of 284 *Mycoplasma pneumoniae* isolates. A) Genetic variability in *M. pneumoniae* populations identified by using a STRUCTURE plot (https://omicsspeaks.com/strplot2). Results show admixture proportions between the fourth and seventh (*K* = 4 and *K* = 7) ancestral populations. Each person is indicated by a vertical line, which is partitioned into *K* = 4 and *K* = 7 colored segments with height proportional to estimated ancestry. Two estimated ancestry majors can be observed in T2–1 clade (blue and red), and 1 estimated ancestry major can be observed in T1–1 clade (dark green). B) Admixture proportions by sampling location. World map shows geographic distribution of admixture proportions from STRUCTURE (*K* = 7) in panel A, which suggests that *M. pneumoniae* populations are related to country. Pie charts are placed at the approximate locations where the strains were isolated, and isolate numbers are indicated by pie chart diameter. Color in pie charts are indicated by the mean composition of 7 ancestral populations. lat., latitude; long., longitude; MLST, multilocus sequence typing.
